# Cognitive Impairment and Non-Invasive Neuromodulation Interventions in Bipolar Disorder: A Narrative Review of Current Status and Future Directions

**DOI:** 10.31083/AP42381

**Published:** 2026-01-12

**Authors:** Guilan Sun, Tongjie Zhuang, Minmin Wang, Xiaomei Zhang

**Affiliations:** ^1^Department of Psychiatry, Huzhou Third Municipal Hospital, The Affiliated Hospital of Huzhou University, 313000 Huzhou, Zhejiang, China; ^2^School of Biomedical Engineering and Instrument Science, Zhejiang University, 310007 Hangzhou, Zhejiang, China

**Keywords:** bipolar disorder, cognitive impairment, non-invasive, neuromodulation

## Abstract

Bipolar disorder (BD) is a severe mental illness characterized by recurrent episodes of mania and depression. The disorder is associated with high rates of relapse and disability, significantly affecting patients’ social functioning and quality of life. It is estimated that 30–50% of individuals with BD do not regain their premorbid level of social functioning, primarily due to persistent cognitive impairments. These cognitive deficits are prominent not only during acute episodes but also persist throughout remission, even when emotional symptoms have stabilized. Multiple studies have demonstrated that cognitive dysfunction is widely recognized as a key predictor of relapse, disease progression, and loss of social functioning in individuals with BD. An increasing body of research suggests that the long-term prognosis of BD is closely linked to cognitive impairment, establishing cognitive remediation as a central therapeutic goal for improving social functioning in this population. However, current pharmacological treatments for cognitive deficits show limited efficacy and are frequently associated with notable side effects. Non-pharmacological approaches, particularly neuromodulation techniques, are increasingly recognized for their potential to improve cognitive deficits in BD. This narrative review summarizes the latest findings on neuromodulation interventions for cognitive impairment in BD, with a focus on the current applications and future directions of non-invasive neuromodulation techniques.

## Main Points

1. Persistent cognitive impairment is a core feature of bipolar disorder (BD), significantly impacting patients’ functional outcomes and quality of life, and it remains present even during periods of stable mood (euthymia).

2. Non-invasive neuromodulation techniques—specifically repetitive transcranial magnetic stimulation (rTMS), transcranial alternating current stimulation (tACS), and transcranial direct current stimulation (tDCS)—show promising potential for improving cognitive deficits in BD, with study demonstrating enhancements in memory, executive function, attention, and verbal fluency.

3. These neuromodulation techniques are generally safe and well-tolerated, with side effects typically being mild and transient, such as scalp discomfort or headache, making them viable adjunctive treatment options.

4. Current evidence is promising but preliminary, highlighting an urgent need for larger, standardized, and long-term study to optimize stimulation parameters, identify precise brain targets, and confirm the sustained efficacy of these interventions for cognitive remediation in BD.

## 1. Introduction

Bipolar disorder (BD) is a severe mental illness characterized by extreme fluctuations in mood, including alternating episodes of mania, hypomania, and depression [1]. During manic or hypomanic episodes, patients typically exhibit elevated mood, racing thoughts, hyperactivity, and impulsive behaviors, whereas depressive episodes are marked by persistent sadness, anhedonia, fatigue, and apathy [2]. BD is classified into two primary subtypes based on the severity of manic symptoms: bipolar I disorder (BD-I), defined by the occurrence of at least one full manic episode, and bipolar II disorder (BD-II) [3], characterized by hypomanic episodes alternating with major depressive episodes [4].

A substantial body of evidence indicates that BD patients frequently experience significant cognitive impairments, including deficits in attention, executive function, verbal memory, working memory, and processing speed [5, 6]. Notably, these cognitive deficits persist not only during acute mood episodes but also during the euthymic phases [7], constituting a core feature of BD [8, 9, 10]. Such impairments profoundly compromise patients’ overall quality of life, occupational functioning, interpersonal relationships, and daily executive performance [11, 12], while simultaneously increasing suicide risk [13]. The global lifetime prevalence of BD is estimated at 1–2%, with BD-I affecting approximately 1.0–1.6% and BD-II affecting 0.5–2.0% of the population [14]. BD typically emerges during adolescence, with a mean onset age of 18–25 years. While BD-I shows comparable incidence rates between males and females, BD-II is slightly more prevalent in females. The disorder exhibits significant familial aggregation, with heritability estimates of 10% to 20% [15]. Furthermore, BD is frequently comorbid with other psychiatric conditions, such as anxiety disorders, substance use disorders, and attention-deficit/hyperactivity disorder [16, 17], complicating diagnosis, treatment, and exacerbating functional impairments and long-term prognosis.

Earlier conceptualizations of BD posited that patients achieved full functional recovery, encompassing cognitive domains, during euthymic phases once affective symptoms stabilized. However, accumulating evidence over the past 20 years has fundamentally challenged this perspective. It is now well-established that a significant proportion of euthymic BD patients exhibit persistent cognitive dysfunction [18, 19], particularly in areas such as executive function and memory, even in the absence of acute mood symptomatology [20, 21]. This recognition marks a pivotal shift in the nosology and understanding of BD. Crucially, this pattern distinguishes BD from schizophrenia, where cognitive deficits are typically more pervasive and enduring across all illness phases. Consequently, the notion of complete inter-episode cognitive recovery in BD is increasingly considered outdated. Current evidence positions cognitive impairment as a chronic, core feature of BD, persisting across mood states including euthymia, and representing a critical determinant of functional outcome [9, 22]. This evolving understanding is strongly supported by meta-analytic evidence showing significant cognitive deficits during euthymia, alongside study documenting impairments in executive function and memory in euthymic cohorts [23].

Current therapeutic strategies for cognitive impairment in BD primarily include pharmacological treatments [24], psychotherapy [25], and neuromodulation techniques. However, pharmacological treatments are often limited by suboptimal efficacy, poor tolerability, and adverse effects. In contrast, non-invasive neuromodulation approaches—such as repetitive transcranial magnetic stimulation (rTMS), transcranial alternating current stimulation (tACS), and transcranial direct current stimulation (tDCS)—have demonstrated promising potential in improving cognitive deficits in BD patients. This section reviews the current status of interventions for cognitive impairment in BD, with a focus on emerging non-invasive neuromodulation therapies and their future prospects.

## 2. Method

To search the study investigating non-invasive neuromodulation interventions for cognitive impairment in BD, We systematically searched PubMed (https://pubmed.ncbi.nlm.nih.gov/), Web of Science (https://www.webofscience.com), and PsycINFO electronic databases (https://www.apa.org/pubs/databases/psycinfo). The search encompassed articles published between January 2006 and 2025, with all retrieved publications limited to the English language. Some searching terms were preferred. They were as follows: “bipolar disorder” and “cognitive impairment” and “repetitive transcranial magnetic stimulation”, “BD” and “executive dysfunction” and “rTMS”, “bipolar disorder” and “memory deficit” and “tDCS”, “BD” AND “attention deficit” and “tACS”, “bipolar disorder” and “neuromodulation”, alongside related terminology combinations. Meanwhile, all clinical investigations involving adolescent and adult BD patients (aged ≥15 years), irrespective of comorbidity status (e.g., anxiety disorders, substance use; see Section 1) or mood phase (euthymic/acute), were considered for inclusion. This current review incorporated original research across methodological designs, ranging from single-case reports and open-label trials to randomized controlled trials (RCTs). Additionally, relevant systematic reviews and meta-analyses were included to contextualize mechanistic and clinical discussion points.

## 3. Manifestations of Cognitive Impairment in BD

### 3.1 Executive Dysfunction

Executive functions, a set of higher-order cognitive processes regulated by the prefrontal cortex, encompass inhibitory control, planning and organizational abilities, and task-switching flexibility. These functions enable individuals to modulate behavior, adapt to complex environments, and adjust actions based on goals and contextual feedback. Impairments in executive functions significantly compromise daily living skills, occupational performance, and social functioning in patients [12].

Inhibitory Control: During manic episodes, patients exhibit exacerbated impulsive behaviors, including reckless decision-making, risk-taking, and deficits in behavioral inhibition. These manifestations are associated with functional abnormalities in the prefrontal cortex, particularly regions responsible for inhibitory control and decision-making [26].

Deficits in Planning and Organization: Patients with BD demonstrate marked difficulties in planning and executing complex tasks, especially during depressive or euthymic phases. Neuroimaging study suggests that reduced prefrontal cortical activity underlies these deficits, impairing long-term planning and efficient information organization [23].

Task-Switching Impairments: Patients show reduced cognitive flexibility in switching tasks or managing multiple tasks, which may be associated with impaired task-switching abilities. This deficit hinders their ability to flexibly respond to dynamic situations [23].

The neural basis of executive dysfunction in BD is closely linked to structural and functional abnormalities in the brain. Magnetic resonance imaging (MRI) study reveals volume reductions and aberrant functional connectivity in key regions such as the prefrontal cortex, cingulate gyrus, and amygdala, which are critical for executive regulation [24]. Additionally, dysregulation of neurotransmitter systems, particularly dopamine and glutamate, has been implicated as a biological mechanism underlying these deficits.

### 3.2 Memory Impairments

Memory dysfunction is another prevalent cognitive deficit in BD, characterized by impairments in short-term memory, working memory, and long-term memory. Short-term memory deficits manifest as patients struggle to retain and manipulate information over brief periods, particularly during complex tasks, which impedes multitasking performance [27]. Working memory, a critical component of the memory system, is essential for temporarily storing and processing task-relevant information. Impairments in this domain disrupt planning, problem-solving, and decision-making abilities [28]. Long-term memory involves both episodic memory (the ability to recall personal experiences or events) and semantic memory (the retention of general knowledge and facts). Episodic memory impairments hinder the recollection of autobiographical events [29], while semantic memory deficits affect access to factual knowledge [30]. Collectively, these impairments compromise patients’ capacity to encode, retrieve, and update information, thereby impacting daily functioning and social interactions. Neuroimaging evidence identifies hippocampal atrophy as a key neural correlate of these memory deficits [31, 32].

The study has demonstrated that deficits in working memory are prevalent in BD, particularly during manic and depressive episodes, exerting significant impacts on cognitive performance and social functioning. BD patients exhibit varying degrees of impairment in both semantic memory (long-term retention of general knowledge and information) and episodic memory (recall of specific events or contextual details). Notably, while semantic memory is relatively preserved, episodic memory is more prominently impaired in BD patients, especially during periods of affective instability [33]. Prospective memory, which involves remembering future plans and tasks, is also significantly affected in BD. Research indicates that patients struggle to execute planned activities in daily life, reflecting diminished prospective memory capacity, particularly in scenarios requiring complex information processing. These memory impairments substantially compromise social and occupational functioning. Deficits in working and prospective memory contribute to workplace inefficiencies, distractibility, and difficulties in task completion. Additionally, everyday memory challenges may hinder interpersonal relationship maintenance and adherence to treatment regimens.

### 3.3 Deficits in Attention and Information Processing Speed

BD exhibit significant attention impairments, primarily manifested as difficulty sustaining attention, inadequate attention allocation, and reduced focus maintain. During task execution, patients are easily distracted and struggle to concentrate on complex or attention-demanding tasks requiring sustained focus. These deficits in attention negatively impact occupational performance, leading to reduced work efficiency, frequent errors, and challenges in multitasking. Additionally, slowed information processing speed reduces patient’s responsiveness to complex tasks, hindering their ability to adapt to rapidly changing environments. Such cognitive impairments may exacerbate the risk of affective symptom relapse, as diminished cognitive function increases life stressors and interpersonal difficulties [34]. Notably, BD patients demonstrate pronounced deficits in sustained attention and information processing speed, particularly during depressive episodes. These impairments manifest as difficulty in task focus and delayed information processing, posing significant challenges in work environments requiring rapid decision-making and prolonged concentration [23].

Information processing speed refers to an individual’s ability to efficiently process information within a limited timeframe. BD patients exhibit marked reductions in information processing speed across various disease phases. The study indicates that even during euthymic phases, their processing speed remains lower than that of healthy controls. This decline not only affects cognitive performance but also impairs social functioning, such as reaction speed and task completion in daily and occupational settings [35].

### 3.4 Impairments in Verbal Fluency and Language Skills

BD patients exhibit deficits in speech production, verbal organization, lexical retrieval, syntactic coherence, and language comprehension. Verbal fluency—defined as the speed, organization, and flexibility of language expression—is particularly affected during manic and depressive episodes. During manic phases, speech is often rapid, disorganized, and tangential, a phenomenon described as “flight of ideas”. Conversely, during depressive episodes, speech becomes slowed and reduced in volume. These fluency deficits may reflect impairments in executive functions, such as planning and initiating speech [20]. Research shows that even in remission, BD patients exhibit persistent verbal fluency deficits, particularly in speech generation and organization. Verbal fluency tests, such as timed category word listing, are commonly used to assess these impairments. The study demonstrates that BD patients perform significantly worse on such tasks compared to healthy controls [22]. Furthermore, these fluency deficits are closely associated with dysfunction in the prefrontal cortex (PFC), a critical region for language production and executive control. Language impairments extend beyond fluency to include higher-order cognitive-linguistic processes, such as vocabulary use, syntactic structuring, and comprehension. BD patients exhibit marked difficulties in lexical retrieval, grammatical construction, and understanding complex language. These issues are linked to deficits in working memory, executive function, and attention—core cognitive domains essential for proficient language skills. Notably, language impairments differ across affective states: manic episodes may involve disorganized syntax and verbose speech, while depressive episodes are characterized by impoverished vocabulary and simplified sentence structures. Such symptoms underscore the depletion of cognitive resources during language processing in BD.

Notably, improving psychosocial functioning and quality of life is a paramount goal in BD treatment, with cognitive remediation positioned as a central clinical target [4]. Extensive evidence suggests that cognitive impairment not only mediates adverse psychosocial outcomes but also predicts unfavorable employment prospects, directly impacting occupational stability and long-term functional recovery [36]. Importantly, persistent cognitive dysfunction persists in a substantial proportion of BD patients even after symptom remission, exacerbating psychological burdens and increasing direct/indirect economic costs of disease management. From a global health perspective, BD contributes significantly to disability-adjusted life years (DALYs), underscoring its profound societal impact [37].

Given the centrality of cognitive impairment in BD, interventions must adopt intensive and sustained strategies. Beyond traditional symptom management, comprehensive cognitive assessment, monitoring, and rehabilitation are imperative. Multimodal therapeutic approaches are essential to enhance cognitive capacity, thereby improving overall quality of life and mitigating long-term disease consequences. Consequently, developing and implementing targeted cognitive remediation programs for BD patients represents both an urgent research priority and a critical step toward optimizing treatment efficacy and reducing societal burden.

## 4. Current Status of Neuromodulation in Treating Cognitive Impairments

A summary of key studies investigating these techniques is provided in Table 1 (Ref. [38, 39, 40, 41, 42, 43, 44, 45]).

**Table 1.** **Summary of study on neuromodulation techniques for cognitive impairment in Bipolar Disorder**.

Authors	Type of study	Stimulation frequency	Phase of BD	Results
Yang LL *et al*. (2019) [38]	Randomized Controlled Trial (RCT)	High-frequency rTMS	Euthymic Phase	Improved cognitive functions (Wechsler Memory Scale scores), no significant adverse effects.
Sciortino D *et al*. (2021) [39]	Systematic Review of RCTs	Varied (mostly high-frequency)	Euthymic Phase	rTMS enhances cognitive performance in euthymic BD patients, limited efficacy during acute depressive episodes.
Yin M *et al*. (2020) [40]	RCT	High-frequency rTMS	Stroke-related Cognitive Impairment	Enhanced cognitive function and brain activity, improved performance in daily living tasks.
Haller N *et al*. (2020) [41]	RCT	40 Hz gamma tACS	Major Depression	Reduced depression scores (HAMD, BDI), improved verbal fluency.
Mardani P *et al*. (2023) [42]	RCT	tDCS	BD-I	Improved problem-solving, cognitive reappraisal abilities.
McClintock SM *et al*. (2020) [43]	International RCT	tDCS	Bipolar Depression	Enhanced verbal learning, processing speed, and working memory, influenced by BDNF and COMT gene polymorphisms.
Kuo HI *et al*. (2013) [44]	Neurophysiological Study	High-Definition tDCS	General Population	Induced cortical plasticity, improvements in cognitive function with specific electrode placement.
Zengin G *et al*. (2022) [45]	Review on TMS in Treatment-Resistant BD	Varied (20 Hz rTMS studied)	Treatment-resistant BD	TMS demonstrated safety and efficacy in BD with treatment-resistant depression.

rTMS, repetitive transcranial magnetic stimulation; BD, Bipolar Disorder; tACS, transcranial alternating current stimulation; HAMD, Hamilton Depression Rating Scale; BDI, Beck Depression Inventory; BD-I, bipolar I disorder; tDCS, transcranial direct current stimulation; BDNF, brain-derived neurotrophic factor; COMT, catechol-O-methyltransferase.

### 4.1 Repetitive Transcranial Magnetic Stimulation (rTMS)

rTMS operates on principles of electromagnetic induction, generating induced currents in cortical neurons via magnetic fields. This alters neuronal action potentials and modulates cortical activity, with stimulation frequency (low: ≤1 Hz or high: ≥5 Hz) determining inhibitory or excitatory effects on targeted brain regions. In BD, rTMS mechanisms involve modulating cortical excitability, enhancing neuroplasticity, and regulating neurotrophic factors. Repetitive stimulation can regulate neurotransmitter metabolism, improve local cerebral blood flow, optimize neuronal microenvironments, and facilitate neural repair [46]. rTMS potentially restore cognitive deficits and spatial memory by modifying sodium/potassium channel dynamics (activation, inactivation, reactivation), thereby enhancing neuronal excitability and hippocampal function.

Cognitive impairment in BD has emerged as a key focus for rTMS interventions. Yang and colleagues [38] conducted a randomized controlled trial (RCT) that demonstrated that 10 days of high-frequency active rTMS significantly improved cognitive functions, such as Wechsler Memory Scale scores, in 52 euthymic BD patients, with no significant adverse effects reported. A systematic review by Sciortino and colleagues [39] incorporating three RCTs indicated that rTMS enhances cognitive performance in euthymic BD patients but shows limited efficacy on cognitive functions during acute depressive episodes, without necessarily affecting mood symptoms. Another RCT reported significant improvements in verbal learning following rTMS [1]. A study administering 20 sessions of 10 Hz rTMS to the left dorsolateral prefrontal cortex (DLPFC) revealed increased low-frequency amplitude in the left medial prefrontal cortex and enhanced functional connectivity between the right medial prefrontal cortex and right ventral anterior cingulate cortex. These changes correlated with improved cognitive performance and quality of life, highlighting rTMS’s capacity to modulate specific neural circuits [40]. High-frequency rTMS may further restore cognitive function by enhancing synaptic signaling, promoting neuroregeneration, and correcting dysfunctions in limbic and local neural systems [47]. Potential mechanisms include upregulated neurogenesis and activation of the brain-derived neurotrophic factor (BDNF) or tropomyosin receptor kinase B (TrkB) pathway, which facilitates functional recovery. Post-rTMS increases in neurotrophic factors (e.g., in the hippocampus) further support its role in neural repair and regeneration [48].

The DLPFC, a hub in the default mode and central executive networks, is a primary target for rTMS due to its critical role in cognition. High-frequency rTMS over the left DLPFC improves attention, working memory [49], executive function [40], and verbal abilities in Alzheimer’s disease (AD) patients [50]. Ljubisavljevic and colleagues and colleagues [51] observed increased relative cerebral blood flow in the left temporo-hippocampal region after 20 Hz rTMS in healthy subjects, indicating that rTMS may enhances cognition via hemodynamic modulation in memory-related circuits.

### 4.2 Transcranial Alternating Current Stimulation (tACS)

tACS, a non-invasive tool in cognitive neuroscience, employs sinusoidal, biphasic currents to entrain neuronal firing and precisely modulate cellular effects [52]. This low-intensity technique avoids sensory stimulation by using weak alternating currents to modulate neural synchronization/desynchronization, thereby regulating cortical excitability and endogenous oscillations. Brain oscillations—rhythmic patterns at specific frequencies—are closely tied to functional states. tACS restores local neural oscillations while synchronizing endogenous rhythms to fixed stimulation frequencies, correcting aberrant oscillatory activity. By coupling or decoupling oscillations across brain regions, tACS enhances inter-regional communication, improving cognition and behavior. It also modulates synaptic calcium influx, induces long-term plasticity. Additionally, it alters neurotransmitter release (e.g., serotonin), contributing to mood regulation [53].

Wang and colleagues [54] evaluated 10 Hz-tACS in 32 patients with unipolar depression, randomized into 10 Hz, 40 Hz, or sham stimulation groups (40 min/day for 5 days). At 2 weeks post-intervention, both active groups showed reduced Montgomery-Åsberg Depression Rating Scale (MADRS) scores, with greater improvements in the 10 Hz group, suggesting tACS’s dual benefits for mood and cognition [54]. Haller *et al*. [41] applied 40 Hz gamma-tACS to depressive patients, observing declines in Hamilton Depression Rating Scale (HAMD) and Beck Depression Inventory (BDI) scores alongside enhanced verbal fluency, indicating cognitive restoration. However, most tACS study focus on healthy populations. For instance, alpha-frequency (10 Hz) tACS over bilateral prefrontal cortices enhances alpha oscillations, improving figural and verbal creativity [55], while 40 Hz tACS boosts logical reasoning via frequency-specific neural resonance. Theta-frequency (4–8 Hz) tACS targeting the frontal lobe improves working memory in older adults by promoting theta phase-amplitude coupling and cross-frontotemporal synchronization [56]. Further clinical trials are needed to validate these findings.

### 4.3 Transcranial Direct Current Stimulation (tDCS)

tDCS applies low-intensity, constant current (1–2 mA) to modulate cortical neuronal activity. Rather than directly inducing neuronal firing, it alters membrane potentials via weak currents delivered through electrodes, thereby adjusting neuronal excitability thresholds and enhancing functional connectivity, particularly in limbic regions like the cingulate cortex [57]. Neuroplasticity—essential for learning and memory—is pathologically altered in BD. tDCS induces short-term functional changes and long-term cortical excitability shifts by influencing N-methyl-D-aspartate receptor (NMDAR) and voltage-gated calcium channels. This triggers synaptic depolarization or hyperpolarization, activating enzymatic cascades that strengthen or weaken synaptic connections. Such synaptic and gene expression changes may underlie tDCS’s neurobiological mechanisms in BD cognitive remediation [58].

Recent study increasingly supports tDCS for BD-related cognitive deficits. Mardani and colleagues [42] conducted an RCT in 30 BD-I patients demonstrated that adjunctive tDCS improved problem-solving and cognitive reappraisal abilities. McClintock and colleagues [43] confirmed tDCS efficacy in enhancing neurocognitive functions (e.g., verbal learning, processing speed, working memory) in bipolar depression, with treatment responses influenced by interactions with BDNF and catechol-O-methyltransferase (COMT) gene polymorphisms. Kuo and colleagues [44] investigated electrode size and placement, showing that tDCS-induced NMDAR modulation drives diverse synaptic plasticity forms, ultimately improving cognition. The DLPFC remains the most common tDCS target. Anodal stimulation over the left DLPFC enhances naming ability, executive function, and attention in Alzheimer’s Disease (AD), while combined with cognitive training, it improves language fluency, executive control, and mood in Parkinson’s disease (PD). Additionally, it has demonstrated beneficial effects in the treatment of post-stroke depression. Anodal tDCS over the ipsilateral DLPFC post-stroke improves attention, working memory, and logical reasoning. Bilateral DLPFC tDCS activates prefrontal-frontal and prefrontal-striatal connectivity, enhancing cognition and mood via ventral striatal dopamine release and cortical-limbic pathway modulation. Functional near-infrared spectroscopy (fNIRS) reveals tDCS-induced local cerebral blood flow changes and remote network effects. Mechanisms may involve calcium signaling, dopamine and γ-aminobutyric acid modulation, and NMDAR-mediated synaptic plasticity.

## 5. Safety of Neuromodulation Techniques

### 5.1 Repetitive Transcranial Magnetic Stimulation (rTMS)

rTMS is generally considered a relatively safe therapeutic modality, with side effects mostly limited to mild and transient discomfort, most commonly headache and localized scalp irritation. Numerous study has demonstrated favorable efficacy and safety profiles for rTMS, including its use in special populations such as adolescents and perinatal women, with no reports of serious adverse events [45]. However, rare cases of seizure induction have been documented, necessitating caution or contraindication in patients with epilepsy [59]. While low-frequency and high-frequency rTMS protocols differ in their mechanisms of action, their overall safety profiles are comparable, with a consistently low risk of seizure induction. Limited evidence suggests a potential risk of inducing manic episodes, requiring further investigation [59].

### 5.2 Transcranial Alternating Current Stimulation (tACS)

tACS modulates neural network activity by regulating the synchrony of neural oscillations. Common adverse effects include mild scalp discomfort, visual flickering, and headache [60]. To date, no severe adverse events have been reported in clinical applications of tACS. Preliminary study suggests its safety in special populations, such as pregnant individuals, though current evidence is limited by small sample sizes and the absence of standardized treatment protocols. The long-term safety of repeated tACS sessions remains underexplored and requires further validation.

### 5.3 Transcranial Direct Current Stimulation (tDCS)

tDCS is associated with mild and manageable side effects, primarily transient scalp tingling, headache, and skin erythema [61]. Compared to rTMS, tDCS presents minimal seizure risk compared to rTMS, positioning it as a safer intervention. Repeated applications of tDCS across multiple sessions have confirmed its safety profile. Nonetheless, isolated reports suggest a potential risk of triggering manic episodes [62], highlighting the need for cautious clinical monitoring.

## 6. Concluding Insights and Future Horizons

In recent years, non-invasive neuromodulation techniques have gained widespread adoption in the treatment of psychiatric disorders due to their painless nature, minimal adverse effects, and demonstrated efficacy, establishing them as one of the most promising medical technologies of the 21st century. Despite their therapeutic potential, current clinical applications remain exploratory, with most study focusing on short-term outcomes and a paucity of long-term follow-up data. The heterogeneous etiology of cognitive impairment in BD, coupled with non-standardized technical protocols, contributes to variability in treatment responses. Future research should prioritize elucidating the mechanisms by which neuromodulation improves cognitive deficits, identifying optimal stimulation targets (e.g., via Functional Magnetic Resonance Imaging (fMRI) or fNIRS-guided localization), and defining individualized protocols for stimulation parameters (intensity, frequency), intervention timing, session frequency, and treatment duration. Large-scale, multicenter randomized controlled trials are essential to validate the long-term efficacy and safety of these techniques in ameliorating cognitive dysfunction in BD.

This review aims to provide clinicians and researchers with updated insights into the application of neuromodulation technologies for cognitive impairment in BD and to explore their potential as adjunctive therapeutic strategies in future clinical practice.

## References

[1] McIntyre RS, Berk M, Brietzke E, Goldstein BI, López-Jaramillo C, Kessing LV (2020). Bipolar disorders. *Lancet (London, England)*.

[2] Tondo L, Vázquez GH, Baldessarini RJ (2017). Depression and Mania in Bipolar Disorder. *Current Neuropharmacology*.

[3] Budde M, Forstner AJ, Adorjan K, Schaupp SK, Nöthen MM, Schulze TG (2017). Genetics of bipolar disorder. *Der Nervenarzt*.

[4] Grande I, Berk M, Birmaher B, Vieta E (2016). Bipolar disorder. *Lancet (London, England)*.

[5] Bora E (2018). Neurocognitive features in clinical subgroups of bipolar disorder: A meta-analysis. *Journal of Affective Disorders*.

[6] Dickinson T, Becerra R, Coombes J (2017). Executive functioning deficits among adults with Bipolar Disorder (types I and II): A systematic review and meta-analysis. *Journal of Affective Disorders*.

[7] Lima IMM, Peckham AD, Johnson SL (2018). Cognitive deficits in bipolar disorders: Implications for emotion. *Clinical Psychology Review*.

[8] Kopf J, Glöckner S, Althen H, Cevada T, Schecklmann M, Dresler T (2023). Neural Responses to a Working Memory Task in Acute Depressed and Remitted Phases in Bipolar Patients. *Brain Sciences*.

[9] Sadana D, Gupta RK, Jain S, Kumaran SS, G S R, Thennarasu K (2019). Neurocognitive profile of patients with Bipolar Affective Disorder in the euthymic phase. *Asian Journal of Psychiatry*.

[10] Razavi MS, Fathi M, Vahednia E, Ardani AR, Honari S, Akbarzadeh F (2024). Cognitive rehabilitation in bipolar spectrum disorder: A systematic review. *IBRO Neuroscience Reports*.

[11] Koene J, Zyto S, van der Stel J, van Lang N, Ammeraal M, Kupka RW (2022). The relations between executive functions and occupational functioning in individuals with bipolar disorder: a scoping review. *International Journal of Bipolar Disorders*.

[12] Liu X, Ma X, Wang W, Zhang J, Sun X, Luo X (2021). The functional impairment of different subtypes and occupational states in euthymic patients with bipolar disorder. *BMC Psychiatry*.

[13] Miller JN, Black DW (2020). Bipolar Disorder and Suicide: a Review. *Current Psychiatry Reports*.

[14] Clemente AS, Diniz BS, Nicolato R, Kapczinski FP, Soares JC, Firmo JO (2015). Bipolar disorder prevalence: a systematic review and meta-analysis of the literature. *Revista Brasileira De Psiquiatria (Sao Paulo, Brazil: 1999)*.

[15] Scott K, Nunes A, Pavlova B, Meier S, Alda M (2023). Familial traits of bipolar disorder: A systematic review and meta-analysis. *Acta Psychiatrica Scandinavica*.

[16] Sesso G, Brancati GE, Masi G (2023). Comorbidities in Youth with Bipolar Disorder: Clinical Features and Pharmacological Management. *Current Neuropharmacology*.

[17] Preuss UW, Schaefer M, Born C, Grunze H (2021). Bipolar Disorder and Comorbid Use of Illicit Substances. *Medicina (Kaunas, Lithuania)*.

[18] Samamé C, Martino DJ, Strejilevich SA (2012). Social cognition in euthymic bipolar disorder: systematic review and meta-analytic approach. *Acta Psychiatrica Scandinavica*.

[19] Samamé C, Martino DJ, Strejilevich SA (2015). An individual task meta-analysis of social cognition in euthymic bipolar disorders. *Journal of Affective Disorders*.

[20] Robinson LJ, Ferrier IN (2006). Evolution of cognitive impairment in bipolar disorder: a systematic review of cross-sectional evidence. *Bipolar Disorders*.

[21] Little B, Flowers C, Blamire A, Thelwall P, Taylor JP, Gallagher P (2024). Multivariate brain-cognition associations in euthymic bipolar disorder. *Bipolar Disorders*.

[22] Strawbridge R, Carter R, Saldarini F, Tsapekos D, Young AH (2021). Inflammatory biomarkers and cognitive functioning in individuals with euthymic bipolar disorder: exploratory study. *BJPsych Open*.

[23] Bora E, Pantelis C (2015). Meta-analysis of Cognitive Impairment in First-Episode Bipolar Disorder: Comparison With First-Episode Schizophrenia and Healthy Controls. *Schizophrenia Bulletin*.

[24] Soncin S, Brien DC, Coe BC, Marin A, Munoz DP (2016). Contrasting emotion processing and executive functioning in attention-deficit/hyperactivity disorder and bipolar disorder. *Behavioral Neuroscience*.

[25] Valdivieso-Jiménez G (2023). Efficacy of cognitive behavioural therapy for bipolar disorder: A systematic review. *Revista Colombiana De Psiquiatria*.

[26] Cipriani G, Danti S, Carlesi C, Cammisuli DM, Di Fiorino M (2017). Bipolar Disorder and Cognitive Dysfunction: A Complex Link. *The Journal of Nervous and Mental Disease*.

[27] Muralidharan K, Kozicky JM, Bücker J, Silveira LE, Torres IJ, Yatham LN (2015). Are cognitive deficits similar in remitted early bipolar I disorder patients treated with lithium or valproate? Data from the STOP-EM study. *European Neuropsychopharmacology: the Journal of the European College of Neuropsychopharmacology*.

[28] D’Esposito M, Postle BR (2015). The cognitive neuroscience of working memory. *Annual Review of Psychology*.

[29] Staniloiu A, Markowitsch HJ (2020). Episodic memory is emotionally laden memory, requiring amygdala involvement. *The Behavioral and Brain Sciences*.

[30] Haubrich J, Bernabo M, Baker AG, Nader K (2020). Impairments to Consolidation, Reconsolidation, and Long-Term Memory Maintenance Lead to Memory Erasure. *Annual Review of Neuroscience*.

[31] Borders AA, Ranganath C, Yonelinas AP (2022). The hippocampus supports high-precision binding in visual working memory. *Hippocampus*.

[32] Pereira LP, Köhler CA, de Sousa RT, Solmi M, de Freitas BP, Fornaro M (2017). The relationship between genetic risk variants with brain structure and function in bipolar disorder: A systematic review of genetic-neuroimaging study. *Neuroscience and Biobehavioral Reviews*.

[33] Vrabie M, Marinescu V, Talaşman A, Tăutu O, Drima E, Micluţia I (2015). Cognitive impairment in manic bipolar patients: important, understated, significant aspects. *Annals of General Psychiatry*.

[34] Solé B, Jiménez E, Torrent C, Reinares M, Bonnin CDM, Torres I (2017). Cognitive Impairment in Bipolar Disorder: Treatment and Prevention Strategies. *The International Journal of Neuropsychopharmacology*.

[35] Tsitsipa E, Fountoulakis KN (2015). The neurocognitive functioning in bipolar disorder: a systematic review of data. *Annals of General Psychiatry*.

[36] Baune BT, Li X, Beblo T (2013). Short- and long-term relationships between neurocognitive performance and general function in bipolar disorder. *Journal of Clinical and Experimental Neuropsychology*.

[37] Catalá-López F, Gènova-Maleras R, Vieta E, Tabarés-Seisdedos R (2013). The increasing burden of mental and neurological disorders. *European Neuropsychopharmacology: the Journal of the European College of Neuropsychopharmacology*.

[38] Yang LL, Zhao D, Kong LL, Sun YQ, Wang ZY, Gao YY (2019). High-frequency repetitive transcranial magnetic stimulation (rTMS) improves neurocognitive function in bipolar disorder. *Journal of Affective Disorders*.

[39] Sciortino D, Pigoni A, Delvecchio G, Maggioni E, Schiena G, Brambilla P (2021). Role of rTMS in the treatment of cognitive impairments in Bipolar Disorder and Schizophrenia: a review of Randomized Controlled Trials. *Journal of Affective Disorders*.

[40] Yin M, Liu Y, Zhang L, Zheng H, Peng L, Ai Y (2020). Effects of rTMS Treatment on Cognitive Impairment and Resting-State Brain Activity in Stroke Patients: A Randomized Clinical Trial. *Frontiers in Neural Circuits*.

[41] Haller N, Senner F, Brunoni AR, Padberg F, Palm U (2020). Gamma transcranial alternating current stimulation improves mood and cognition in patients with major depression. *Journal of Psychiatric Research*.

[42] Mardani P, Javdani H, Zolghadriha A, Mousavi SE, Dadashi M (2023). A Randomized Clinical Trial to Assess the Effect of Medication Therapy Plus tDCS on Problem-solving and Emotion Regulation of Patients with Bipolar Disorder Type I. *Clinical Psychopharmacology and Neuroscience: the Official Scientific Journal of the Korean College of Neuropsychopharmacology*.

[43] McClintock SM, Martin DM, Lisanby SH, Alonzo A, McDonald WM, Aaronson ST (2020). Neurocognitive effects of transcranial direct current stimulation (tDCS) in unipolar and bipolar depression: Findings from an international randomized controlled trial. *Depression and Anxiety*.

[44] Kuo HI, Bikson M, Datta A, Minhas P, Paulus W, Kuo MF (2013). Comparing cortical plasticity induced by conventional and high-definition 4 × 1 ring tDCS: a neurophysiological study. *Brain Stimulation*.

[45] Zengin G, Zulkif Topak O, Atesci O, Culha Atesci F (2022). The efficacy and safety of transcranial magnetic stimulation in treatment-resistant bipolar depression. *Psychiatria Danubina*.

[46] Lefaucheur JP, André-Obadia N, Antal A, Ayache SS, Baeken C, Benninger DH (2014). Evidence-based guidelines on the therapeutic use of repetitive transcranial magnetic stimulation (rTMS). *Clinical Neurophysiology: Official Journal of the International Federation of Clinical Neurophysiology*.

[47] Zheng Y, Mao YR, Yuan TF, Xu DS, Cheng LM (2020). Multimodal treatment for spinal cord injury: a sword of neuroregeneration upon neuromodulation. *Neural Regeneration Research*.

[48] Luo J, Zheng H, Zhang L, Zhang Q, Li L, Pei Z (2017). High-Frequency Repetitive Transcranial Magnetic Stimulation (rTMS) Improves Functional Recovery by Enhancing Neurogenesis and Activating BDNF/TrkB Signaling in Ischemic Rats. *International Journal of Molecular Sciences*.

[49] Liu Y, Yin M, Luo J, Huang L, Zhang S, Pan C (2020). Effects of transcranial magnetic stimulation on the performance of the activities of daily living and attention function after stroke: a randomized controlled trial. *Clinical Rehabilitation*.

[50] Cotelli M, Calabria M, Manenti R, Rosini S, Zanetti O, Cappa SF (2011). Improved language performance in Alzheimer disease following brain stimulation. *Journal of Neurology, Neurosurgery, and Psychiatry*.

[51] Ljubisavljevic MR, Javid A, Oommen J, Parekh K, Nagelkerke N, Shehab S (2015). The Effects of Different Repetitive Transcranial Magnetic Stimulation (rTMS) Protocols on Cortical Gene Expression in a Rat Model of Cerebral Ischemic-Reperfusion Injury. *PloS One*.

[52] Elyamany O, Leicht G, Herrmann CS, Mulert C (2021). Transcranial alternating current stimulation (tACS): from basic mechanisms towards first applications in psychiatry. *European Archives of Psychiatry and Clinical Neuroscience*.

[53] Pan R, Ye S, Zhong Y, Chen Q, Cai Y (2023). Transcranial alternating current stimulation for the treatment of major depressive disorder: from basic mechanisms toward clinical applications. *Frontiers in Human Neuroscience*.

[54] Wang H, Wang K, Xue Q, Peng M, Yin L, Gu X (2022). Transcranial alternating current stimulation for treating depression: a randomized controlled trial. *Brain: a Journal of Neurology*.

[55] Grabner RH, Krenn J, Fink A, Arendasy M, Benedek M (2018). Effects of alpha and gamma transcranial alternating current stimulation (tACS) on verbal creativity and intelligence test performance. *Neuropsychologia*.

[56] Reinhart RMG, Nguyen JA (2019). Working memory revived in older adults by synchronizing rhythmic brain circuits. *Nature Neuroscience*.

[57] Chase HW, Boudewyn MA, Carter CS, Phillips ML (2020). Transcranial direct current stimulation: a roadmap for research, from mechanism of action to clinical implementation. *Molecular Psychiatry*.

[58] Stagg CJ, Antal A, Nitsche MA (2018). Physiology of Transcranial Direct Current Stimulation. *The Journal of ECT*.

[59] Miron JP, Feffer K, Cash RFH, Derakhshan D, Kim JMS, Fettes P (2019). Safety, tolerability and effectiveness of a novel 20 Hz rTMS protocol targeting dorsomedial prefrontal cortex in major depression: An open-label case series. *Brain Stimulation*.

[60] Antal A, Alekseichuk I, Bikson M, Brockmöller J, Brunoni AR, Chen R (2017). Low intensity transcranial electric stimulation: Safety, ethical, legal regulatory and application guidelines. *Clinical Neurophysiology: Official Journal of the International Federation of Clinical Neurophysiology*.

[61] Nitsche MA, Cohen LG, Wassermann EM, Priori A, Lang N, Antal A (2008). Transcranial direct current stimulation: State of the art 2008. *Brain Stimulation*.

[62] Dondé C, Neufeld NH, Geoffroy PA (2018). The Impact of Transcranial Direct Current Stimulation (tDCS) on Bipolar Depression, Mania, and Euthymia: a Systematic Review of Preliminary Data. *The Psychiatric Quarterly*.

